# Assignment of Chinook Salmon (*Oncorhynchus tshawytscha)* Linkage Groups to Specific Chromosomes Reveals a Karyotype with Multiple Rearrangements of the Chromosome Arms of Rainbow Trout (*Oncorhynchus mykiss*)

**DOI:** 10.1534/g3.113.008078

**Published:** 2013-10-29

**Authors:** Ruth B. Phillips, Linda K. Park, Kerry A. Naish

**Affiliations:** * Department of Biological Sciences, Washington State University, Vancouver, Washington 98686-9600; † Center for Reproductive Biology, Washington State University, Pullman, Washington 99164-4236; ‡ Northwest Fisheries Science Center, Seattle, Washington 98112-2097; § School of Aquatic and Fishery Sciences, University of Washington, Seattle, Washington 98195-5020

**Keywords:** cytogenetic map, comparative salmonid chromosome maps

## Abstract

The Chinook salmon genetic linkage groups have been assigned to specific chromosomes using fluorescence *in situ* hybridization with bacterial artificial chromosome probes containing genetic markers mapped to each linkage group in Chinook salmon and rainbow trout. Comparison of the Chinook salmon chromosome map with that of rainbow trout provides strong evidence for conservation of large syntenic blocks in these species, corresponding to entire chromosome arms in the rainbow trout as expected. In almost every case, the markers were found at approximately the same location on the chromosome arm in each species, suggesting conservation of marker order on the chromosome arms of the two species in most cases. Although theoretically a few centric fissions could convert the karyotype of rainbow trout (2N = 58–64) into that of Chinook salmon (2N = 68) or *vice versa*, our data suggest that chromosome arms underwent multiple centric fissions and subsequent new centric fusions to form the current karyotypes. The morphology of only approximately one-third of the chromosome pairs have been conserved between the two species.

A whole-genome duplication occurred in an ancestor of salmonid fish sometime within the early Tertiary to the late Cretaceous periods (Allendorf and Thorgaard 1984). As a result, their genome size is twice that of their closest relatives, the Esociformes and Osmeriformes (Gregory *et al.* 2007), and their genomes contain a large number of duplicate genes, some of which exhibit residual tetrasomic inheritance (Allendorf and Danzmann 1997). Most salmonids have karyotypes composed of both metacentric (bi-armed) and acrocentric (uni-armed) chromosomes with a modal range of chromosome arms (NF) of 96–104 (Phillips and Rab 2001), whereas most teleost species, especially freshwater groups such as the Esociformes, have a diploid chromosome number of 48–50, with 24 or 25 acrocentric chromosome pairs and NFs of 48–52 (Mank and Avise 2006). After the whole-genome duplication in the common ancestor of salmonid fish, its karyotype would have ∼100 chromosome arms, which is the number found in all current species of the Pacific salmon and trout (genus *Oncorhynchus*). This observation suggests that karyotype changes within the genus will consist primarily of centric fissions and fusions. Because the chromosome number in Chinook salmon (2N = 68) is higher than that in rainbow trout (2N = 58–64), we would expect that a few centric fissions could convert the rainbow trout karyotype to the Chinook salmon karyotype or *vice versa*.

The karyotype of the Chinook salmon has been characterized as 2N = 68, with 16 pairs of metacentric chromosomes and 18 pairs of acrocentric chromosomes (Simon 1963). The fluorescence banding patterns with quinacrine, DAPI, and CMA3 have been described (Phillips *et al.* 1985), and the locations of the nucleolar organizer (28S rDNA) and 5S rDNA have been determined using silver staining, CMA3 banding, and fluorescence *in situ* hybridization (Phillips *et al.* 1985, 1986, 2005b). The NOR is on a small acrocentric chromosome and the 5S rDNA is distributed on the short arms of 5–8 different acrocentric chromosome pairs. The sex chromosome has been identified as the largest acrocentric chromosome pair in the karyotype (#17) using *in situ* hybridization with two male-specific probes: OtY1 (Stein *et al.* 2001) and GH-Y (Phillips *et al.* 2005b). The Chinook sex chromosome pair contains a band of repetitive DNA in the middle of the long arm of the chromosome arm that stains positively with Quinacrine and DAPI. This band is variable in size and is present on both the X and Y chromosomes. The Y chromosome has a larger short arm than the X, explained by a region where the male-specific OtY1 and GH-Y sequences are found. Genetic mapping of microsatellite loci (Omy7INRA, OMM1077, and OMM3018) confirmed that the long arm of the sex chromosome pair corresponds to the long arm of rainbow trout chromosome Omy15 (RT LG7q) (Williamson *et al.* 2008). Fluorescence *in situ* hybridization using a bacterial artificial chromosome (BAC) containing Omy7INRA confirmed that Omy7INRA maps just below the centromere on the long arm of the sex chromosome pair (Williamson *et al.* 2008).

A genetic linkage map has been constructed for Chinook salmon using microsatellites (Naish *et al.*, pp. 2281–2288, companion article), many of which have also been mapped in rainbow trout Nichols *et al.* 2003; Danzmann *et al.* 2005; Guyomard *et al.* 2006; Rexroad *et al.* 2008). The total number of microsatellite markers mapped in Chinook salmon is 361 compared to ∼3000 in rainbow trout. The rainbow trout genetic linkage groups have been assigned to specific trout chromosomes using fluorescence *in situ* hybridization with rainbow trout BACs containing type I and microsatellite markers from the genetic map as probes (Phillips *et al.* 2006). These BACs have also been included in the integration of the linkage map with the physical map (Palti *et al.* 2004), which is available online at Clemson University. This physical map is based on Hind III fingerprinting of BAC clones from three different BAC libraries each constructed from the DNA of a YY male from the Swanson clonal line (Palti *et al.* 2004). We report the assignment and orientation of linkage groups to each chromosome pair in Chinook salmon and the comparison of the resulting Chinook salmon chromosome map with that of rainbow trout (Phillips *et al.* 2006).

## Materials and Methods

### Animal materials

Chinook salmon were originally obtained from the Dworshak, Idaho, spring Chinook strain in 2002.

### BAC library screening

The clones were obtained from two different trout BAC libraries, one made from the rainbow trout OSU XX clonal line (Phillips *et al.* 2003) and the other made from the Swanson YY clonal line (Palti *et al.* 2004). Physical maps for rainbow trout using these BAC libraries are available at www.genome.clemson.edu/physicalmaps/rainbow trout. Initially, filters from the Oregon State University library were screened for type I clones using ^32^P-labeled cloned probes, and later the PCR super-pools from both the Ohio State University and the Swanson libraries were screened for clones containing additional type I loci and microsatellite loci using specific PCR primers for each locus.

### *In situ* hybridization and karyotyping

Chromosome preparations were obtained from Chinook salmon blood by methods described previously (Phillips *et al.* 2006). Briefly, the buffy coat was isolated from whole blood and placed in MEM media with pen-strep, L-glutamine, 10% fetal calf serum, and 200 ug/ml LPS and cultured for 6 days at 20°. Cells were collected by centrifugation and re-suspended in 0.075 M KCl for 30 min, then fixed in 3:1 methanol:acetic acid. Cell suspensions were placed onto clean slides and allowed to dry on a slide warmer with humidity at 40°. BAC DNA was isolated from clones containing a microsatellite marker with a known position in the rainbow trout linkage map using the QIAGEN Plasmid Midi Kit (catalog #12143) following the manufacturer’s protocol. BAC DNA was labeled with either spectrum orange (Vysis) or digoxigenin (Roche), as recommended by the manufacturers. Hybridization with fluorochrome-labeled dUTPs was as suggested by the manufacturer (Vysis) with minor modifications (Phillips *et al.* 2006). Briefly, chromosome preparations were made the day before use and left to dry on a slide warmer at 40° overnight. Just before hybridization, the slides were denatured in a 70% formamide solution at 73° for 5 min. The probe was prepared by adding labeled DNA with human placental DNA and rainbow trout Cot1 DNA (for blocking) to the Vysis hybridization solution and denatured at 73° for 5 min. Hybridization was allowed to proceed under a sealed coverslip in a humidified chamber at 37° overnight. The next day, the slides were washed first with 0.3% NP40 in 0.4× SSC at 73° for 3 min, and then with 0.1% NP40 in 2× SSC at room temperature for 1 min. For digoxigenin probes, antibodies to digoxigenin (1/100 dilution in PBS) were applied, and the slides were incubated at 37° for 45 min, according to manufacturer’s instructions. Primary and secondary antibodies to spectrum orange (1/100 and 1/200 dilution in PBS) were used to amplify the signal in many experiments. Slides were counterstained with DAPI/antifade (Vysis) and then examined using an Olympus BX60 microscope and photographed with either a Sensys or a Jai digital camera. Images were captured with Cytovision software (Applied Imaging) and selected karyotypes were prepared using Genus software (Applied Imaging). Chromosome pairs were identified using relative size and bands revealed by DAPI staining and chromosome arm ratios. Chromosomes were assorted according to size using the software described and adjustments made by hand to conform to the standard chromosome arm ratios and DAPI staining patterns. In some cases, dual hybridizations with two different BAC clones containing markers from specific linkage groups (in two different colors) were performed to confirm these assignments.

### Comparison of Chinook salmon and rainbow trout linkage maps and karyotypes

We examined several genetic maps for rainbow trout (Nichols *et al.* 2003; Danzmann *et al.* 2005; Guyomard *et al.* 2006; Rexroad *et al.* 2008) for markers found on the Chinook salmon genetic maps (see companion article). Two of the rainbow trout maps (Nichols *et al.* 2003; Guyomard *et al.* 2006) contain 30 linkage groups corresponding to 60 chromosomes, whereas the other rainbow trout genetic maps (Danzmann *et al.* 2005; Rexroad *et al.* 2008) contain 29 linkage groups corresponding to 58 chromosomes. Interior rainbow trout strains usually have 58 chromosomes, whereas coastal strains can have from 60 to 64 chromosomes (Thorgaard 1983). It has previously been shown that LG 4 and LG 25, from the map of Nichols *et al.* (2003), which correspond to acrocentric chromosome pairs 25 (LG 4) and 29 (LG 25) in the Oregon State University rainbow trout strain, are fused to form a metacentric chromosome pair in the strains with 58 chromosomes (Phillips *et al.* 2005a). Therefore, in the genetic linkage maps based on rainbow trout with 58 chromosomes, LG 4 is not present as a separate linkage group but rather is found as part of LG 25 as expected from the cytogenetic data. This same fusion has been found in Chinook salmon (this article). For the comparison with Chinook salmon, we used the linkage groups from the rainbow trout strain with 60 chromosomes and 30 linkage groups. However, all rainbow trout genetic maps were searched for genetic markers found on Chinook salmon genetic maps.

## Results

**Figure 1:**
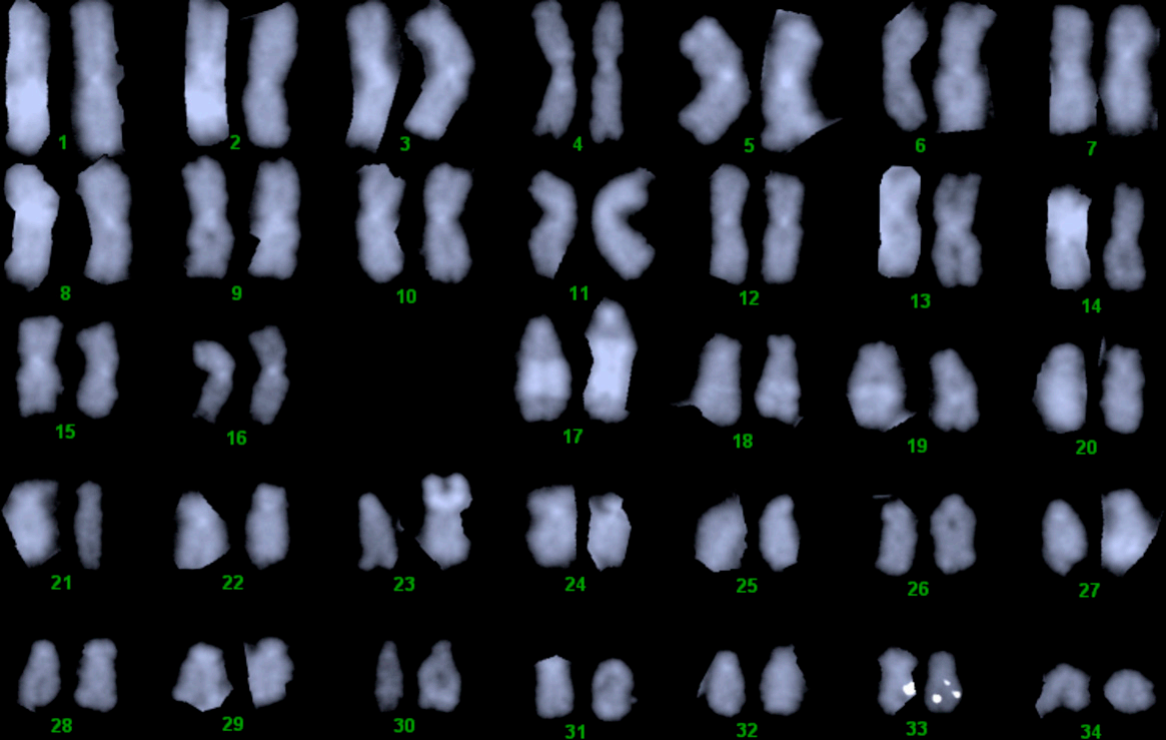
Hybridization of a BAC containing genetic marker SCAR163 from the rainbow trout sex chromosome to Ots 33. The Chinook sex chromosome pair is Ots 17 in the karyotype.

Hybridization experiments were performed with rainbow trout BAC clones containing genetic markers mapped to each Chinook salmon linkage group as probes. These BAC clones had been previously hybridized to rainbow trout chromosomes. In some cases, dual hybridizations were performed to confirm chromosome fusions. Figure 1 shows the result of hybridization with a BAC containing a genetic marker (SCAR163) mapped to the long arm of the rainbow trout sex chromosome (Omy Sex), which hybridizes to the long arm of Chinook chromosome 33 (Ots33, Ck LG19). The Chinook sex chromosome is the largest acrocentric (pair #17) with the large interstitial DAPI bright band in the middle of the long arm, so these results confirm that the sex chromosomes are different in the two species (Stein *et al.* 2001; Phillips *et al.* 2005b). A composite of images showing the results of hybridizations of one probe for each linkage group is shown in Supporting Information, Figure S1. In each case, the sex chromosome pair from the same metaphase spread is shown below the chromosome containing the probe signal to indicate its relative size. The sex chromosome was used because it is the largest acrocentric in the karyotype and is readily identified because it has an interstitial band of repetitive DNA of variable size that is usually visible with DAPI staining (Stein *et al.* 2001).

**Figure 2:**
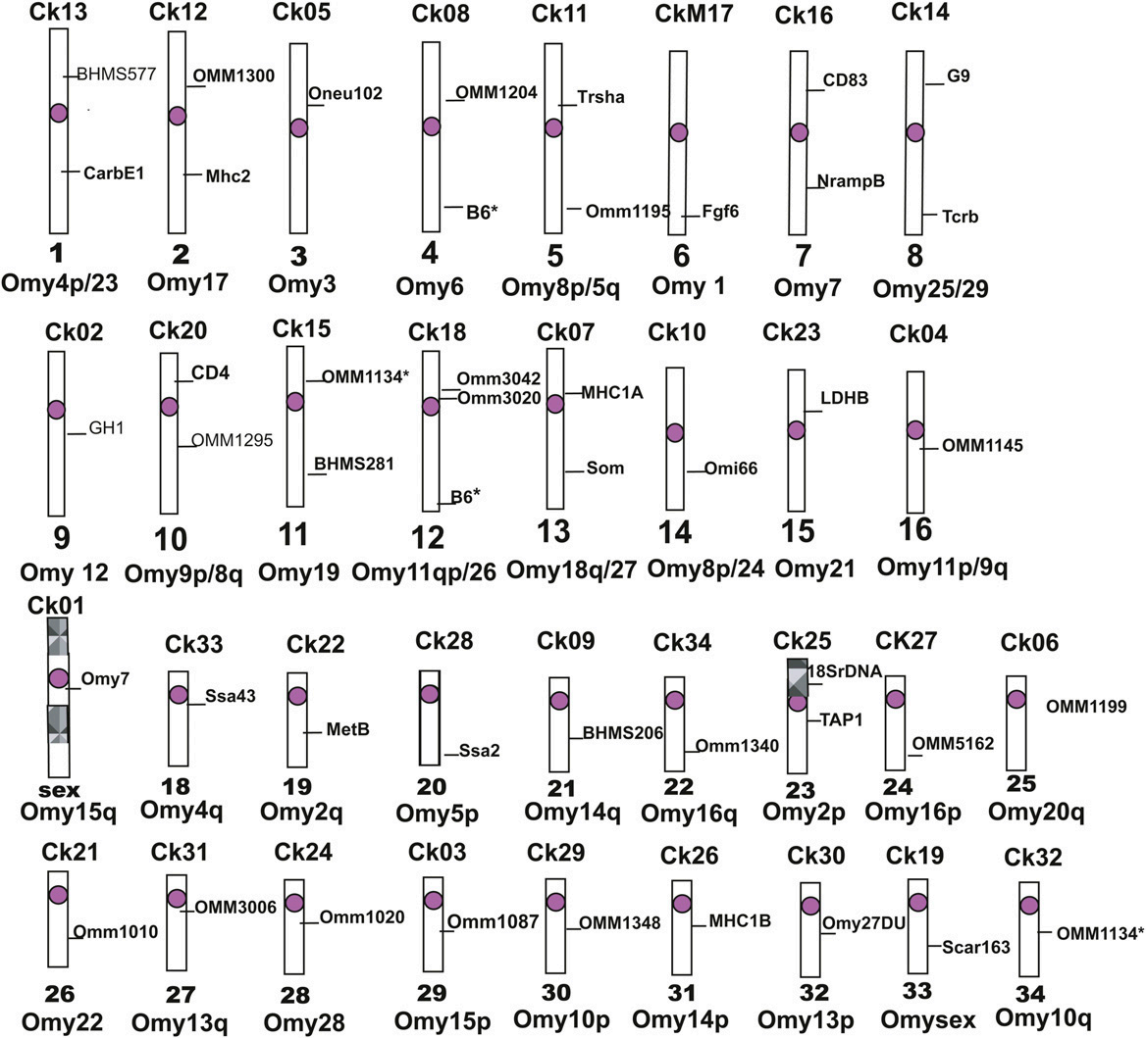
An ideogram of the Chinook salmon karyotype showing location of probes mapped by *in situ* hybridization.

The BAC clones used to assign the genetic linkage groups to chromosomes are shown in Table 1 with the Chinook chromosome arm assignment and linkage group and corresponding rainbow trout chromosome arm and linkage group. An ideogram of the Chinook salmon karyotype showing the location of the BAC probes used to assign linkage groups to chromosomes and indicating the corresponding rainbow trout chromosome arms is shown in Figure 2. An ideogram showing the Chinook salmon karyotype and LGs with corresponding rainbow trout chromosome arms and LGs in color (without the genetic markers) is available in Figure S2.

**Table 1:** Assignment of Chinook genetic linkage groups to chromosomes

Chromosome	Arm	CK LG	Markers	BAC Clone	RT Chromosome	RT LG*^a^*
Ots1	1p	CK13	BHMS577	102N02a	Omy4p	24p
	1q		CarbE1	86E19o	Omy23	30
Ots2	2p	CK12	OMM1300	318G19s	Omy 17	29q
	2q		MHC2	4C02o	Omy 17	29p
Ots3	3p	CK05	Oneu102	18G23a	Omy3p	31p
Ots4	4p	CK8	OMM1204	113I17s	Omy 6p	10p
	4q		B6	B6o	Omy6q	10q
Ots5	5p	CK11	TRSHA	354I19s	Omy8p	23p
	5q		OMM1195	107J7s	Omy5q	8q
Ots6	6q	CK17	FGF6	122J17o	Omy1q	6q
Ots7	7p	CK16	CD83	17c24o	Omy7p	12p
	7q		NrampB	146I11o	Omy7q	12q
Ots8	8p	CK14	G9	G9o	Omy25	4
	8q		TCRb	270C12o	Omy29	25
Ots9	9q	CK02	GH1	167I21o	Omy12q	9q
Ots10	10p	CK20	CD4	275D24s	Omy9p	21p
	10q		OMM1295	146Hs	Omy8q	23q
Ots11	11p	CK15	OMM1134*^a^*	271M12s	Omy19p	14p
	11q		BHMS281	198E23a	Omy19q	14q
Ots12	12p	CK18	OMM3042	C9o	Omy11q	19p
	12p		OMM3020	F1o	Omy11p	19p
	12q		B6*^a^*	B6o	Omy26	18
Ots13	13p	CK7	MHCIA	24K3o	Omy18q	16q
	13q		Somatolactin	193J21o	Omy27	11
Ots14	14q	CK10	Omi66	366K10o	Omy24	26
Ots15	15p	CK23	LDHB	176H21s	Omy21p	15p
Ots16	16q	CK04	OMM1145	520I2s	Omy9q	21
Ots17	17q	CKSex	Omy7INRA	197M11s	Omy15q	7q
Ots18		CK33	Ssa43	322O19a	Omy4q	24q
Ots19		CK22	MetB	127C24o	Om2q	27q
Ots20		CK28	Ssa2	88023a	Omy5p	8p
Ots21		CK09	BHMS206	26A22a	Omy14q	3q
Ots22		Ck34	OMM1340	277k4o	Omy16q	22q
Ots23		Ck25	TAP1	34E19o	Omy2p	27p
Ots24		CK27	OMM5162	25G06o	Omy16p	22p
Ots25		CK06	OMM1199	33GD8o	Omy20q	17q
Ots26		CK21	OMM1010	231J20s	Omy22	5
Ots27		Ck31	OMM3006	C11o	Omy13	2q
Ots28		CK24	OMM1020	239K12s	Omy28	13
Ots29		Ck03	OMM1087	340I3s	Omy15p	7p
Ots30		CK29	OMM1348	117A19s	Omy10p	20p
Ots31		CK26	MHC1B	20C13o	Omy14p	3p
Ots32		CK30	Omy27DU	118G14o	Omy13p	2p
Ots33		CK19	SCAR163	171H7s	SEX	1
Ots34		CK32	OMM1134*^a^*	271M12s	10q	20q

Chromosomes are listed from the largest to the smallest, with Ots1–16 being metacentric (bi-armed) chromosomes and Ots 17–34 being acrocentric (uni-armed) chromosomes. Linkage groups correspond to those on the genetic map in the companion article (Naish *et al.* 2013). In the future, the linkage groups will be renamed to correspond to the chromosome numbers. B6 was a random clone isolated from the RT library, which goes to two telomeres. Ots 18–34 are single-armed chromosomes. a, Atlantic salmon library (CHORIO); o, Oregon State University BAC library; s, Swanson BAC library; p, short arm of the metacentric (bi-armed) chromosomes; q, long arm of the metacentric (bi-centric) chromosomes.

a The rainbow trout LGs were used before the genetic map was connected to the chromosomes. In the recent maps (Rexroad *et al.* 2008; Palti *et al.* 2004; Phillips *et al.* 2009), the linkage groups are numbered the same as the chromosomes. The p and q include Ots1–Ots16 for Chinook salmon and Omy1–Omy22 for rainbow trout. Chinook chromosomes Ots17–Ots34 and rainbow trout chromosomes Omy23–Omy30 are acrocentric single-armed chromosomes. o=OSU library, s=Swanson library, and a=Atlantic salmon CHORIO library. The BACs from the “a” library were previously used as probes on rainbow trout chromosomes.

Large blocks of genetic markers corresponding to whole-chromosome arms in rainbow trout correspond to specific chromosome arms in Chinook salmon (see companion article by Naish *et al.* 2013). There is extensive conservation of synteny in these regions, such that proximal or distal markers are almost always in the same location between the species and usually the order of markers along the chromosomes is very similar. This conclusion is the result of comparison of the position of markers on the genetic maps prepared for rainbow trout by three different authors (Danzmann *et al.* 2005; Guyomard *et al.* 2006; Rexroad *et al.* 2008) with the position of markers on the genetic map of Chinook (companion article by Naish *et al.* 2013) and the comparison of the position of BAC clones containing specific markers from rainbow trout on the chromosome arms of both rainbow trout (Phillips *et al.* 2006) and Chinook salmon in this article. The data in Table 1 list the rainbow trout chromosome arms and linkage groups with the homologous chromosome arms and linkage groups of Chinook salmon. Table 2 groups the homeologous chromosome arms and linkage groups that were identified in the companion article by Naish *et al.* (2013) with the corresponding rainbow trout chromosome arms and linkage groups.

**Table 2:** Homeologies between Chinook salmon linkage groups with corresponding chromosome arms shown in parenthesis

Chinook Linkage Groups and Chromosomes	Chromosomes	Rainbow Trout Duplicated Markers	Ancestral LG
1 and 23 (Ots17 and 15p)	Omy15q and 21p	Omm1077	K
2 and 31 (Ots9q and 27)	Omy12q and 13q	Omm1274, Omm1218	E
8 and 18q (Ots04q and 12))	Omy6q and 26	Omm1202, B6	J
9 and 14 (Ots21 and 08q)	Omy14q and 29	Ogo2 UW	GH
30 and 12 (Ots32 and 02p)	Omy13p and 17q	OMM1167, Omm 1330	L
25 and 5 (Ots23 and 03p)	Omy2p and 3p	Omm1016, Omm5062, OtsG423UCD	B
15 and 32 (Ots11p and 34)	Omy19p and 10q	Omm5017, Omm1657, Omm5106	F
7 and 26 (Ots16q and 3p)	Omy18q and 14p	Omm3066	B

The corresponding rainbow trout chromosome arms and the duplicated markers are also provided.

There was one case (Ots12, CK LG18) in which markers from three rainbow trout chromosome arms (Omy11p, 11q, and 26) were mapped to one Chinook chromosome. Hybridization with BAC clones containing genetic markers confirmed this arrangement. Thus, it appears that an inversion occurred on Omy11 before fusion with Omy 26, so that markers from both Omy11p and Omy11q are found on one arm of this metacentric chromosome in addition to markers from Omy26 on the other arm. One microsatellite marker from Omy11p was also found on Ots16, CK LG4. So, the chromosome arm with the remaining markers from Omy11p appears to have fused with Omy9q to form Ots16, the smallest metacentric chromosome pair. Also, the microsatellite marker order also suggests an apparent inversion on Omy 20 (Ots 25, CK06), such that all of the microsatellite markers were transferred to Omy20q before the fission (companion article by Naish *et al.*, 2013).

## Discussion

Unlike rainbow trout, which vary in chromosome number between 2N of 58 and 2N of 64 (Thorgaard 1983), Chinook salmon have a very stable karyotype of 2N of 68 with NF of 100 chromosome arms in North America and Asia (Simon 1963; Muramoto *et al.* 1974; Phillips *et al.* 1985, 1986; Stein *et al.* 2001). Although rainbow trout have traditionally been assigned an NF of 104, if we ignore the short arm of chromosome 20 (LG17) that is composed almost entirely of ribosomal DNA and consider the smallest metacentric chromosome (Omy22, LG5) in the rainbow trout karyotype as a single arm that underwent a pericentric inversion (*i.e.*, Chr 22p,q), then rainbow trout have 100 chromosome arms as well, as do all the other *Oncorhynchus* species. When the rainbow trout chromosomes are arranged according to size, the smallest metacentric chromosome pair (LG 5) is between the largest and second largest acrocentric chromosomes, a result that supports the inversion hypothesis for Omy22. In addition, rainbow trout Omy20 and Omy22 each correspond to a single chromosome arm in the Atlantic salmon karyotype (Phillips *et al.* 2009) and in the ancestral vertebrates (Danzmann *et al.* 2008; Nakatani *et al.* 2007). When the location of genetic loci mapped in rainbow trout are indicated on the Chinook salmon female genetic map, it can be seen that there is an excellent correspondence between the 50 diploid chromosome arms or equivalent segments in each species, including the order of the markers in most cases. To properly evaluate the similarity in marker order will, of course, require a higher marker density on the Chinook salmon genetic map. In any case, we can conclude that the karyotypes of both species are composed of the same 50 large syntenic blocks, which correspond to extant or previous chromosome arms in the haploid karyotypes. The locations of these syntenic blocks are shown in Table 1 and Figure 2.

There are eight chromosome fusions in the Chinook salmon karyotype of various chromosome arms in the 2N = 60 rainbow trout karyotype to produce new metacentrics in the Chinook karyotype. One of the fusion chromosomes, Ots 8 (Ck LG14), corresponds to Omy25 and Omy29 in the rainbow trout 2N = 60 karyotype and is also present in rainbow trout with the 2N = 58 karyotype (Phillips *et al.* 2005a). This is the ancestral karyotype for rainbow trout, which may explain why it is also found in Chinook. In almost every case, the markers that were localized on the Chinook salmon fusion chromosomes map to a location (proximal or distal portion of the arm) similar to where they were localized on the corresponding rainbow trout chromosome arm. This strongly supports the hypothesis that the fusions were centromere-to-centromere. This is also true for intraspecific chromosome fusions in rainbow trout (R. B. Phillips 2005a, unpublished observations). Given the presence of multiple chromosome fusions in different populations of rainbow trout, perhaps the large number of chromosome rearrangements between rainbow trout and Chinook salmon is not surprising.

Salmonid fishes underwent a period of extensive speciation after the whole-genome duplication of their common ancestor, which probably had a typical teleost karyotype comprising 25 pairs of acrocentric chromosomes (Mank and Avise 2006). Therefore, we would expect that 25 pairs of duplicated segments might be identified in each species. All 25 duplicated chromosome arms have been identified in Atlantic salmon (Lien *et al.* 2011). Twenty-one duplicated segments have been identified in rainbow trout (Phillips *et al.* 2006), but only seven have the support of nine or more markers. So far, eight have been identified in Chinook salmon and these include all of the ones with strong support in rainbow trout. This suggests that as more genetic markers are mapped, more homeologies will be detected in Chinook salmon. The genome duplication is thought to have taken place more than 100 million years ago, but the lineages of rainbow trout and Chinook salmon diverged only approximately six million years ago (Wilson and Turner 2009). It appears that extensive diploidization occurred early on, but some of the duplicated chromosomes have continued to exchange genes, especially at the telomeres. Why the same chromosome arms are still undergoing recombination in both lineages is not known. In rainbow trout, 15–20% of the BAC clones hybridized to two different chromosomes, usually to loci on the homeologous chromosomes; however, in Chinook salmon we found only approximately 10% of BACs hybridized to more than one chromosome pair. Although this might suggest that there has been a reduction in duplicate markers in this species, a denser genetic map will be required to evaluate this hypothesis properly. One explanation for the difference might be that the BACs used in both studies were from rainbow trout, so they might be more likely to cross-hybridize with duplicate loci in the homologous species. Genetic markers on orthologous chromosome arms in rainbow trout and Chinook salmon are usually in the same order, which is not unexpected given the recent divergence between the two species. We would expect that minor inversions would eventually scramble the gene order on the chromosome arms between the two species, as has occurred between zebrafish (*Danio rerio*) and medaka (*Oryzias latipes*), which diverged at least 140 million years ago (Nakatani *et al.* 2007). There was a whole-genome duplication in the early teleost lineage, so that genetic markers on two chromosome pairs in zebrafish and medaka correspond with the same duplicated syntenic blocks in the human genetic map (Nakatani *et al.* 2007). Like most teleosts, these two species have approximately two dozen mainly uni-armed chromosome pairs (Mank and Avise 2006). This similarity in chromosome number is in contrast to chromosome evolution in mammals, in which centric fusions and fissions of chromosome arms are common and have produced a great variety of diploid chromosome numbers. Two chromosome arms in zebrafish (2N = 50) and medaka (2N = 48) (descendants of the teleost whole-genome duplication) can, in most instances, be related to at least four whole or partial chromosomal arms in the salmonid species (Danzmann *et al.* 2008).

Note Added in Proof: See also Kerry A. Naish, Ruth B. Phillips, Marine S. O. Brieuc, Lyndsay R. Newton, Anna E. Elz, and Linda K. Park, 2013 Comparative Genome Mapping Between Chinook Salmon (*Oncorhynchus tshawytscha*) and Rainbow Trout (*O. mykiss*) Based on Homologous Microsatellite Loci G3: Genes, Genomes, Genetics 3: 2281–2288.

## Supplementary Material

Supporting Information
