# Supplementary material for: Assignment of Chinook Salmon (Oncorhynchus tshawytscha) Linkage Groups to Specific Chromosomes Reveals a Karyotype with Multiple Rearrangements of the Chromosome Arms of Rainbow Trout (Oncorhynchus mykiss)
Source: G3 (Bethesda). 2013 Oct 29;3(12):2289–95. doi: 10.1534/g3.113.008078 (PMC3852390; doi:10.1534/g3.113.008078)
Supplement: Supporting Information [file supp_3_12_2289__index.html]

Assignment of Chinook Salmon (Oncorhynchus tshawytscha) Linkage Groups to Specific Chromosomes Reveals a Karyotype with Multiple Rearrangements of the Chromosome Arms of Rainbow Trout (Oncorhynchus mykiss) — Supporting Information 

# Assignment of Chinook Salmon (*Oncorhynchus tshawytscha)* Linkage Groups to Specific Chromosomes Reveals a Karyotype with Multiple Rearrangements of the Chromosome Arms of Rainbow Trout (*Oncorhynchus mykiss*)

## Supporting Information for Phillips, Park, and Naish, 2013

**Files in this Data Supplement:**

- Supporting Information - Figures S1-S2 (PDF, 520 KB)
- Figure S1 - Composite of 34 partial karyotypes showing results of hybridization with BAC clones (labeled in red) containing a marker mapped to each specific linkage group of Chinook salmon. (PDF, 332 KB)
- Figure S2 - Ideogram of the Chinook salmon karyotype showing location of rainbow trout chromosome arms. (PDF, 226 KB)
