# Supplementary material for: Assignment of Chinook Salmon (Oncorhynchus tshawytscha) Linkage Groups to Specific Chromosomes Reveals a Karyotype with Multiple Rearrangements of the Chromosome Arms of Rainbow Trout (Oncorhynchus mykiss)
Source: G3 (Bethesda). 2013 Oct 29;3(12):2289–95. doi: 10.1534/g3.113.008078 (PMC3852390; doi:10.1534/g3.113.008078)
Supplement: Supporting Information [file supp_g3.113.008078_008078SI.pdf]

G3 · Genes | Genomes | Genetics

Assignment of Chinook Salmon (*Oncorhynchus tshawytscha)* linkage groups to specific chromosomes reveals a karyotype with multiple rearrangements of the chromosome arms of rainbow trout (*Oncorhynchus mykiss*)

Ruth B. Phillips[*], Linda K. Park[§] and Kerry A. Naish[†]
[*] Department of Biological Sciences, Washington State University, Vancouver, WA 98686-9600 USA
[*]Center for Reproductive Biology, Washington State University, Pullman, WA 99164-4236 USA
[§]Northwest Fisheries Science Center,725 Montlake Blvd.East, Seattle, WA 98112-2097 USA
[†]School of Aquatic and Fishery Sciences, Box 355020, University of Washington, Seattle, WA, 98195-5020 USA
[*]Corresponding author

Corresponding Author Information:
Ruth B. Phillips
Department of Biological Sciences, 14204 NE Salmon Creek Avenue, Washington State University-Vancouver, Vancouver, WA 98686-9600 USA
phillipsr@vancouver.wsu.edu; phone : 360-546-9505; fax : 360-546-9064

DOI: 10.1534/g3.113.008078

A.

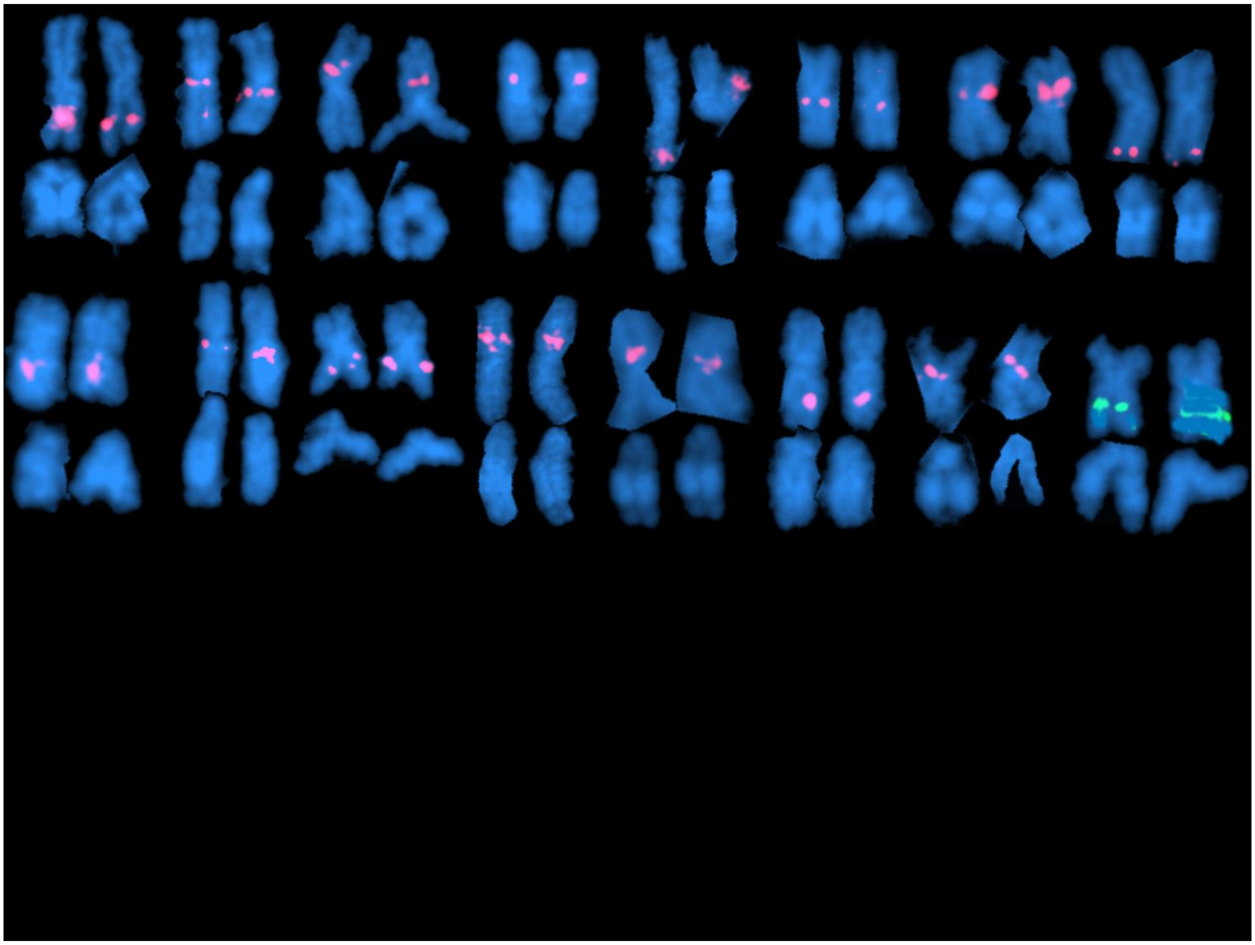

B.

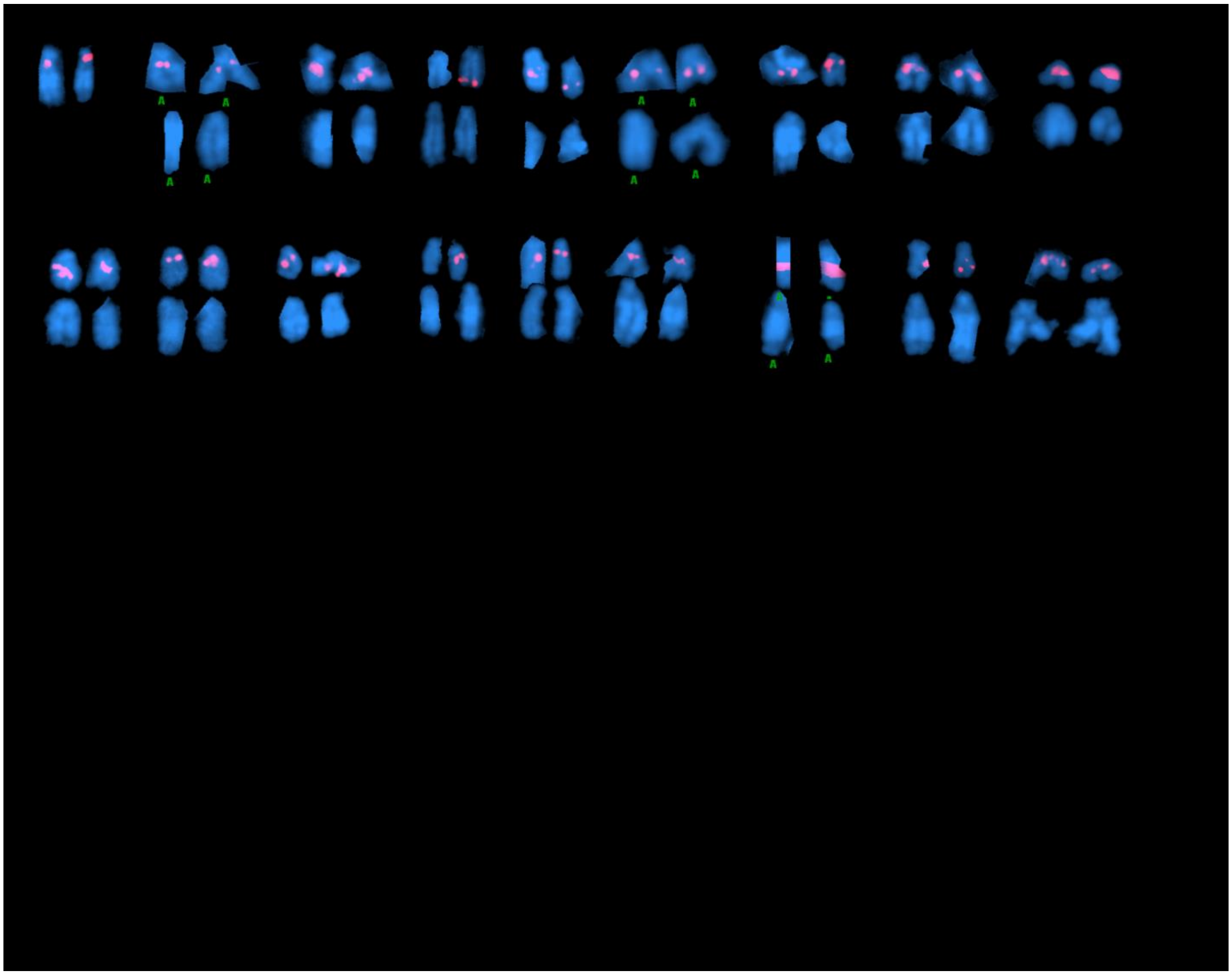

Figure S1 Composite of 34 partial karyotypes showing results of hybridization with BAC clones (labeled in red) containing a marker mapped to each specific linkage group of Chinook salmon. (The BAC clone containing OMM1145 hybridized to Ots 16 was labeled in green). (S1A shows metacentric chromosome pairs and S1B shows acrocentric chromosome pairs.)

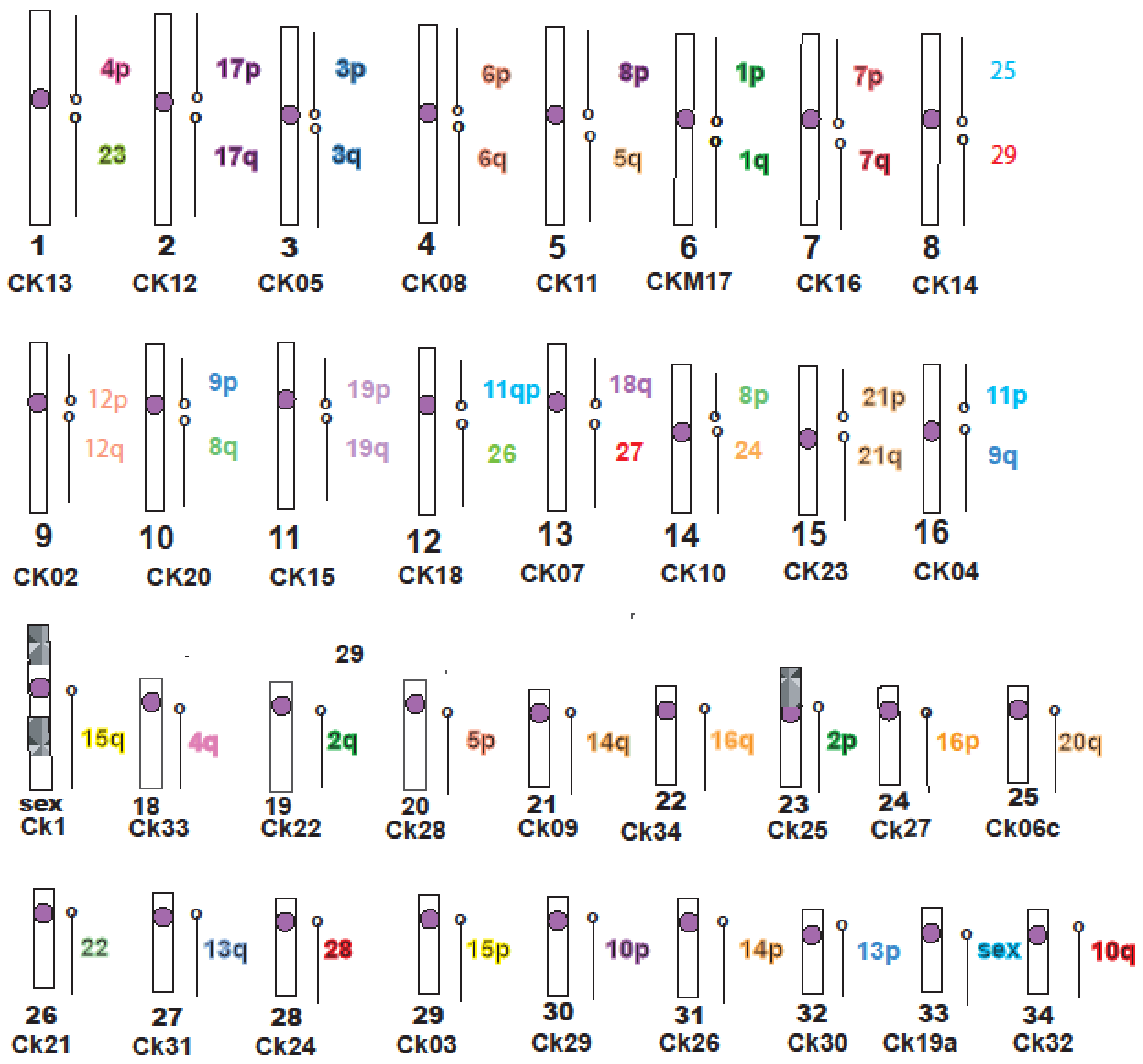

Figure S2 Ideogram of the Chinook salmon karyotype showing location of rainbow trout chromosome arms.
