# Supplementary material for: Assignment of Chinook Salmon (Oncorhynchus tshawytscha) Linkage Groups to Specific Chromosomes Reveals a Karyotype with Multiple Rearrangements of the Chromosome Arms of Rainbow Trout (Oncorhynchus mykiss)
Source: G3 (Bethesda). 2013 Oct 29;3(12):2289–95. doi: 10.1534/g3.113.008078 (PMC3852390; doi:10.1534/g3.113.008078)
Supplement: Supporting Information [file supp_g3.113.008078_FigureS1.pdf]

A.

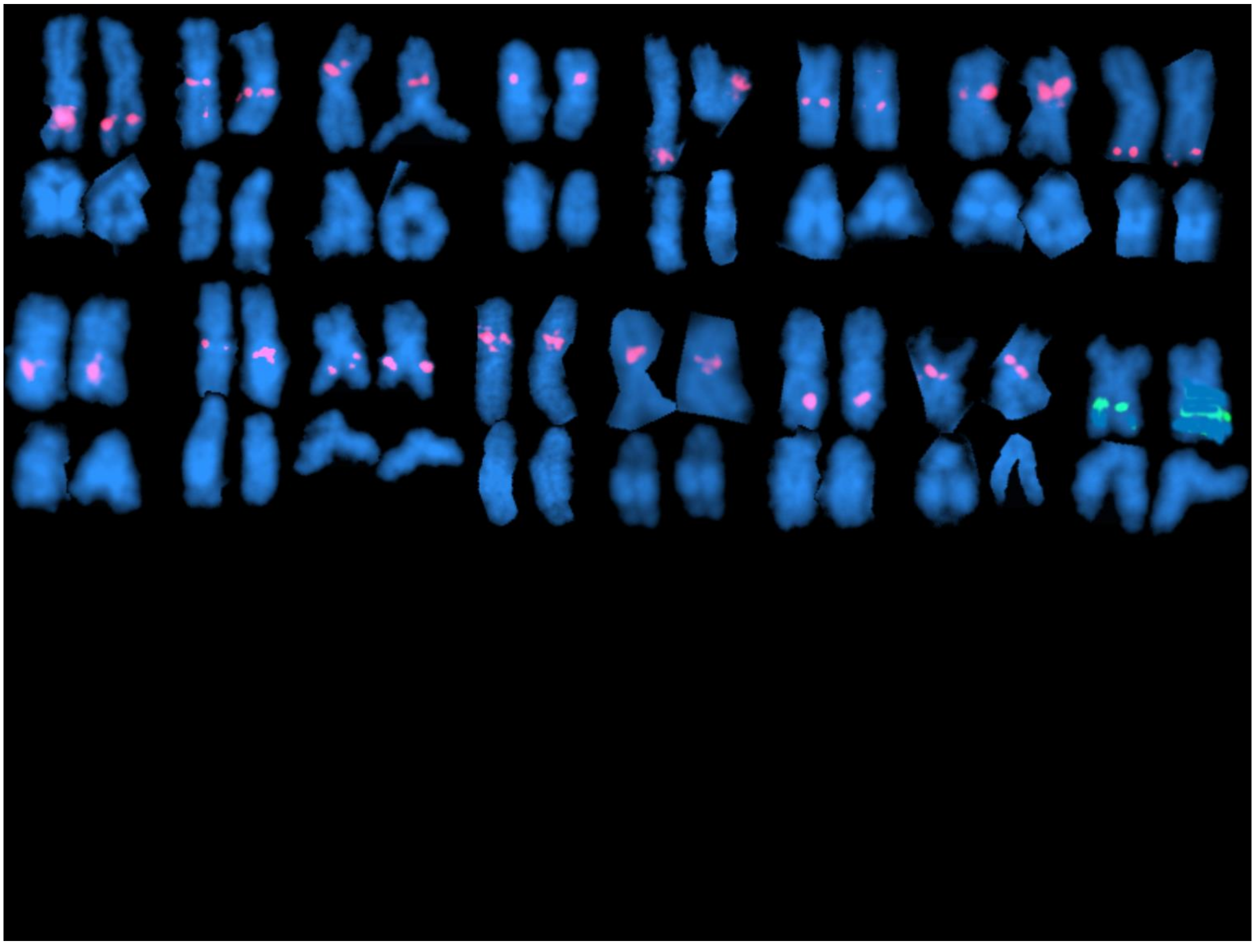

B.

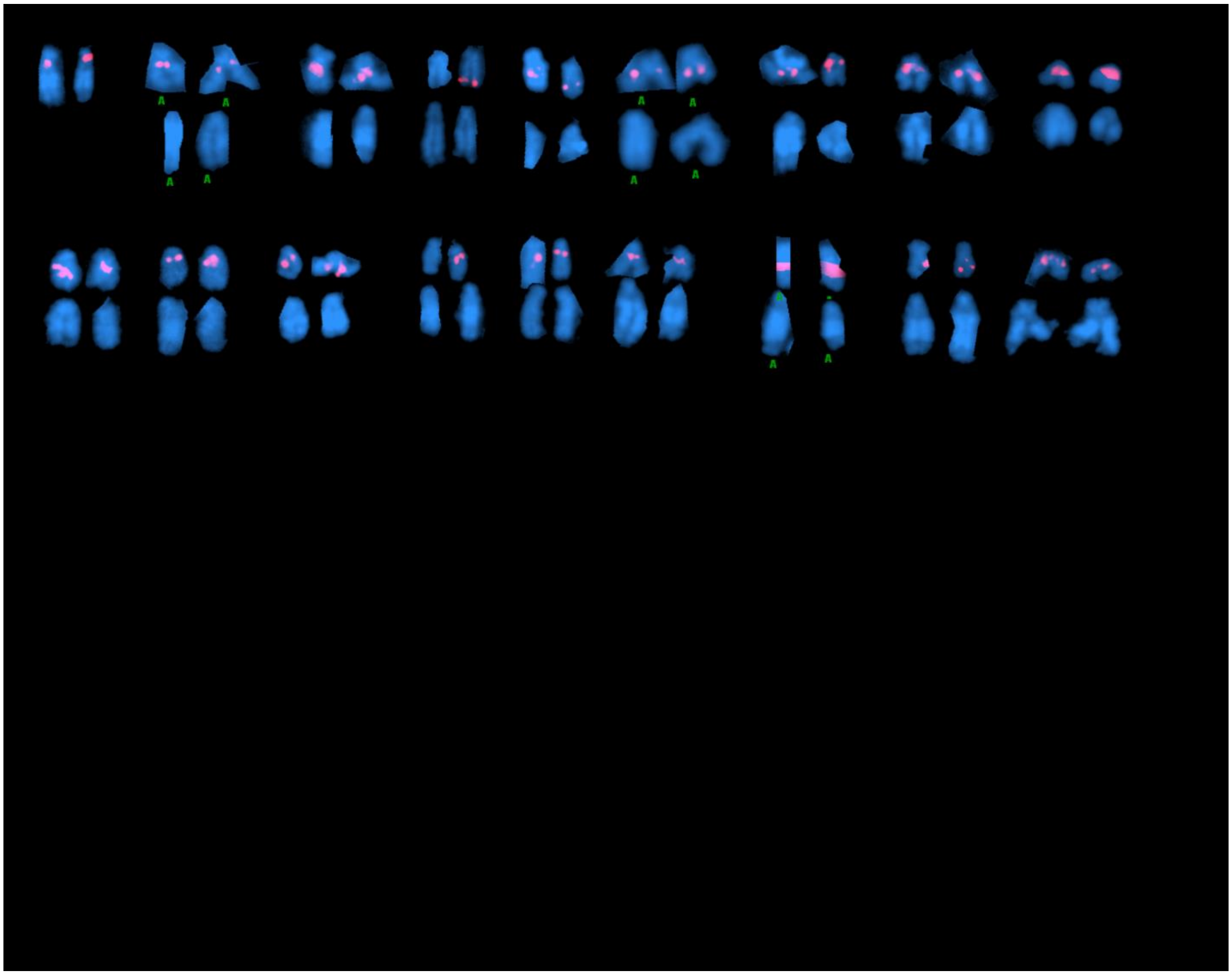

Figure S1 Composite of 34 partial karyotypes showing results of hybridization with BAC clones (labeled in red) containing a marker mapped to each specific linkage group of Chinook salmon. (The BAC clone containing OMM1145 hybridized to Ots 16 was labeled in green). (S1A shows metacentric chromosome pairs and S1B shows acrocentric chromosome pairs.)
