# Supplementary material for: Assignment of Chinook Salmon (Oncorhynchus tshawytscha) Linkage Groups to Specific Chromosomes Reveals a Karyotype with Multiple Rearrangements of the Chromosome Arms of Rainbow Trout (Oncorhynchus mykiss)
Source: G3 (Bethesda). 2013 Oct 29;3(12):2289–95. doi: 10.1534/g3.113.008078 (PMC3852390; doi:10.1534/g3.113.008078)
Supplement: Supporting Information [file supp_g3.113.008078_FigureS2.pdf]

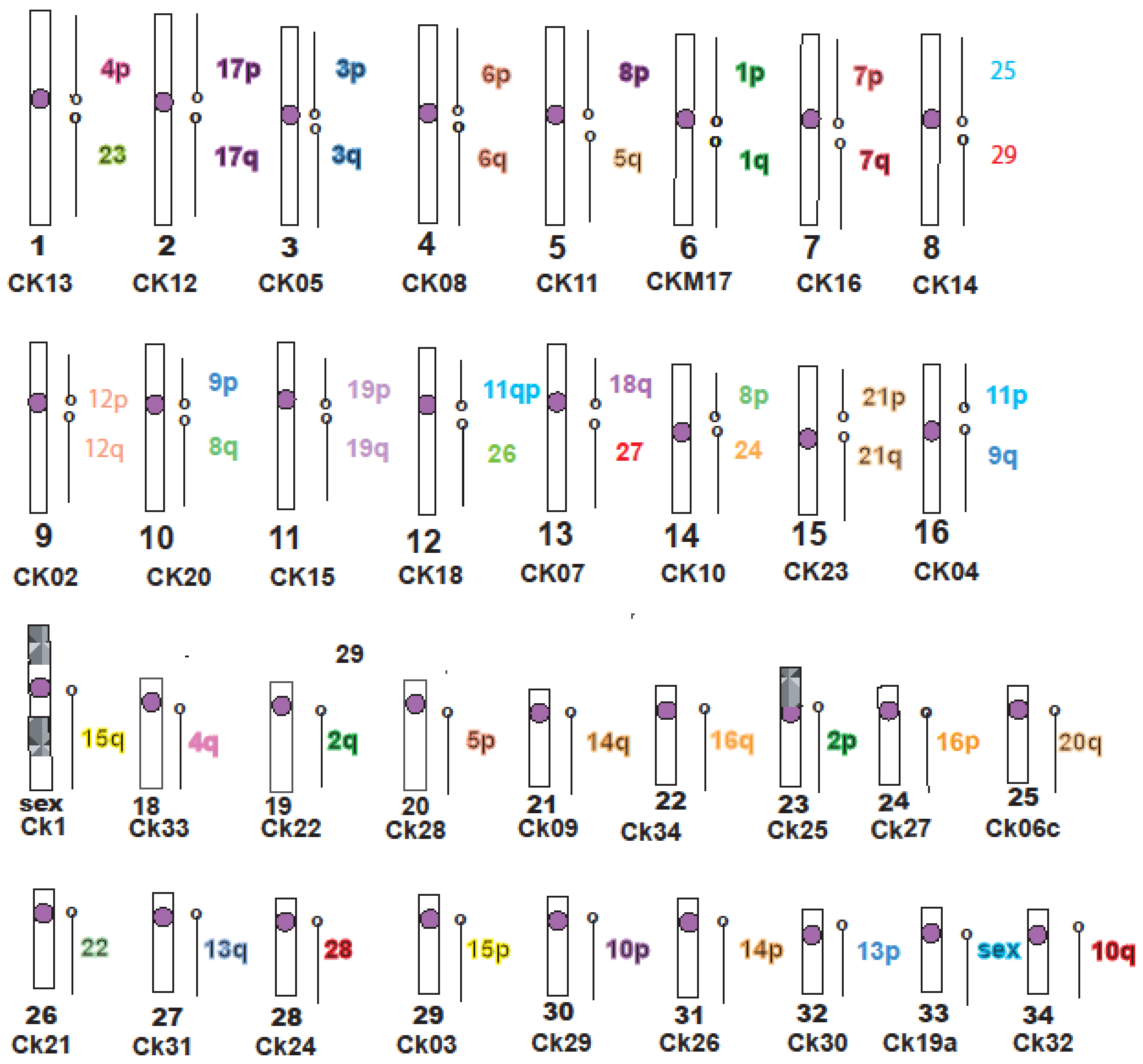

Figure S2 Ideogram of the Chinook salmon karyotype showing location of rainbow trout chromosome arms.
